# Multinuclear Metal Nucleobase Complexes

**DOI:** 10.1155/MBD.1994.241

**Published:** 1994

**Authors:** A. Schreiber, H. Rauter, M. Krumm, S. Menzer, E. C. Hillgeris, B. Lippert

**Affiliations:** 1 Fachbereich Chemie Universität Dortmund Dortmund D -44221 Germany

## Abstract

Of all properties of metal nucleobase complexes, formation of multinuclear species appears to be an outstanding feature. After a brief introduction into well known polymeric metal nucleobase complexes, three aspects recently Studied in our laboratory will be dealt with in more detail: (i) Heteronuclear complexes derived from trans-[(amine)_2_Pt(1-MeC)_2_]^2+^ (1-MeC=1-methylcytosine). They form, e. g. with Pd(II) or Hg(II), upon single deprotonation of the exocyclic amino group of each 1-MeC ligand, compounds of type trans-[(amine)_2_Pt(1-MeC-)_2_MY]^n+^, displaying Pt-M bond formation. (ii) Cyclic nucleobase complexes derived from cis-a_2_Pt(II). A cyclic compound of composition {[(en)Pt(UH-N^1^,N^3^)]_4_}^4+^ (UH=monoanion of unsubstituted uracil) is presented and the analogy with organic calix-[4]-arenes is pointed out. (iii) Cyclic nucleobase complexes from trans-a_2_Pt(II). Possible ways for the preparation of macrocyclic nucleobase complexes containing trans-a_2_Pt(II) linkages are outlined and precursors and intermediates are presented.


### MULTINUCLEAR METAL NUCLEOBASE COMPLEXES

A. Schreiber*, H. Rauter, M. Krumm, S. Menzer, E.C. Hillgeris and B. Lippert

Fachbereich Chemie, Universität Dortmund, D - 44221 Dortmund, Germany

**Abstract**

Of all properties of metal nucleobase complexes, formation of multinuclear species appears to be an outstanding feature. After a brief introduction into well known polymeric metal nucleobase complexes, three aspects recently studied in our laboratory will be dealt with in more detail: (i) Heteronuclear complexes derived from trans-$[(amine)_2Pt(1\text{-}MeC)_2]^{2+}$ (1-MeC=1-methylcytosine). They form, e. g. with Pd(II) or Hg(II), upon single deprotonation of the exocyclic amino group of each 1-MeC ligand, compounds of type trans-$[(amine)_2Pt(1\text{-}MeC^-)_2MY]^{n+}$, displaying Pt-M bond formation. (ii) Cyclic nucleobase complexes derived from cis-$a_2Pt(II)$. A cyclic compound of composition $\{[(en)Pt(UH\text{-}N^1,N^3)]_4\}^{4+}$ (UH=monoanion of unsubstituted uracil) is presented and the analogy with organic calix-[4]-arenes is pointed out. (iii) Cyclic nucleobase complexes from trans-$a_2Pt(II)$. Possible ways for the preparation of macrocyclic nucleobase complexes containing trans-$a_2Pt(II)$ linkages are outlined and precursors and intermediates are presented.

### Introduction

As far as biological aspects of interactions between metal ions and nucleic acids or their constituents, the nucleobases are concerned, many compounds containing a single metal center and a defined number of nucleobase ligands have been prepared and studied[1-4]. In contrast to these, there is at present only little evidence for a role of either polymeric or multinuclear complexes in nucleobase biology. Possible exceptions could be the antitumor active platinum pyrimidine blues, nucleic acid stains such as ruthenium red or the recently described diplatinum compounds of Farrell, which show antitumor activity for the cis- as well as for the trans-isomer[5].

In this paper, we will give a short report on multinuclear metal nucleobase complexes, their binding patterns and the possible application of di- and trinuclear 9-methyladenine (9-MeA) species in metallamacrocycle synthesis. Especially the role of nucleobases, respectively modelnucleobases, as bi- and multidentate ligands is of great importance for the formation of multinuclear compounds. Hereby metal clustering on the nucleobase as a consequence of initial metal binding is a feasibility on the basis of chemical evidence derived from model systems.

### Polymeric Compounds

The polymeric nucleobase complexes known today can be classified in two different groups. The first group, which is well established for many silver compounds, has the nucleobase as a bridging unit being bi- or multidentate (Fig.1). Depending on whether a single metal is involved in complex formation or different metals, there are currently three different binding patterns characterized.

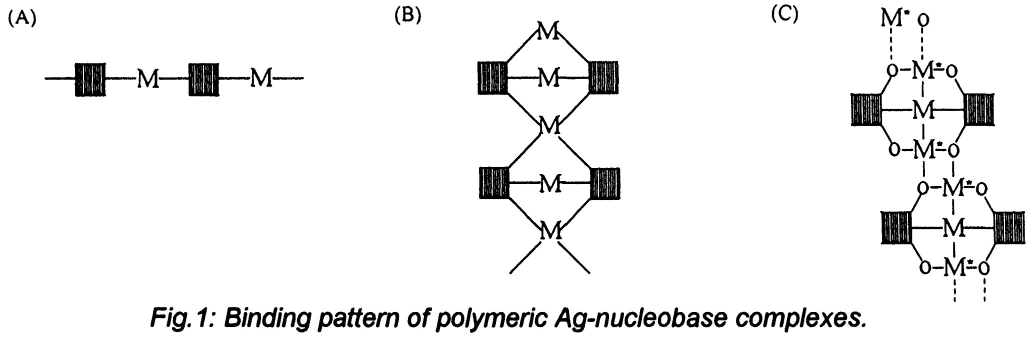

*Fig. 1: Binding pattern of polymeric Ag-nucleobase complexes.*

A simple polymeric chain, with 9-MeA acting as a linking entity between two Ag centers has been prepared by Beauchamp et al [6] (Fig.1a). Figure 1b shows an example for polymeric silver complexes containing the pyrimidine nucleobases 1-methylthymine and 1-methyluracil, respectively, in their anionic forms (1-MeU, 1-MeT). Here, the pyrimidinato ligands are bound to three silver atoms via their carbonyl oxygens and the deprotonated ring nitrogen N(3)[7]. It is evident that the metal centers are inequivalent. One silver, which occupies a crystallographic inversion center, has a linear coordination sphere, whereas the two others display tetrahedral coordination geometries, and are shared between two $Ag(pym)_2$ entities. Figure 1c gives a schematic view of the structure of a polymeric heteronuclear Pt/Ag polymer (M=Pt, M*= Ag)[8]. It is built up of $PtAg_2$-entities which are linked through O(2) and O(4) sites of the uracilato ligands. While the Pt coordination geometry is closely square-planar, the structure reveals a distorted bipyramidal arrangement of ligands around the silver. (Additional contacts to nitrate anions and water oxygens are not shown here).
Referring to the second group of polymers, there are currently a few adducts of mercury dichloride with a variety of guanine derivatives known[9]. In all cases the nucleobase is bound in a monodentate fashion to the mercury with halogen-bridged $HgCl_2$ entities forming the polymeric backbone.

**Heteronuclear complexes**

In extension of our work on multinuclear compounds, we used bis-cytosine-metal compounds as precursors for dinuclear species. In principle, three different possibilities of complex formation are feasible (Fig.2). In the case of (c), metal-metal bond formation can be expected because here the coordination planes are almost perpendicular to each other. This situation facilitates an overlap of the $dz^2$ orbital of M with the $dx^2$-$y^2$ orbital of M` and formation of a M-M` bond[11].

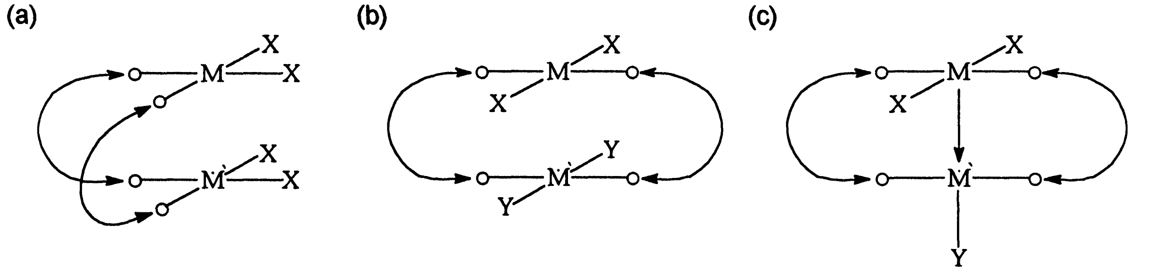

*Fig.2: Possible binding pattern for dinuclear metal nucleobase complexes.*

As has recently been observed by us[11], trans-$[(NH_3)_2Pd(1\text{-}MeC\text{-}N^3)_2]^{2+}$ (1-MeC = neutral 1-methylcytosine), when reacted with trans-$[(NH_3)_2Pd(H_2O)_2]^{2+}$, escapes the steric hindrance between X=Y=$NH_3$ ligands by isomerization and formation of a head / tail cis-$[(NH_3)_2Pd(1\text{-}MeC^-\text{-}N^3,N^4)_2Pd(NH_3)_2]^{2+}$ species instead of the expected trans-compound. According to our findings, the steric clash between X- and Y-ligands is also prevented, with the trans-geometry maintained, if reaction leads to a product as outlined in Fig.2c. Thus reaction of trans-$[(NH_3)_2Pt(1\text{-}MeC\text{-}N^3)_2]^{2+}$ with the diaqua species of trans-Pd(II) yields a dinuclear complex with the coordination planes of both metals in an almost perpendicular arrangement to each other. Pt(II) has a square-pyramidal coordination sphere, whereas Pd(II) is surrounded by its four ligands in a square-planar arrangement. A series of related compounds with different Y ligands has been prepared. The cytosinato ligands ($1\text{-}MeC^-$) are bridging both metal centers through their N(3) and deprotonated N(4) sites. In this case, as well as in the similar heteronuclear species with Pt(II) / Hg(II), which were obtained under analogous conditions, the nucleobases are in a head / head arrangement. The metal-metal bond lengths range from 2.511 (1)-2.515 (4) Å in Pt-Pd complexes and from 2.765 (1)-2.835 (1) Å in Pt-Hg compounds[10-12].

**Cyclic systems**

Another compound, which initiated our interest in metallamacrocycles, is the tetranuclear metal complex $[(en)Pt(UH\text{-}N^1,N^3)]_4^{4+}$(with UH=monoanion of uracil), which can be considered a calix-[4]-arene analogue[13,14]. The bridging entity of the classical calix-[4]-arenes, usually a $CH_2$-group, is here replaced by the (en)Pt(II)-unit. Complex formation in solution through condensation of four $[(en)Pt(UH\text{-}N^1)(H_2O)]^+$ species occurs spontaneously:

$$4[(en)Pt(UH\text{-}N^1)(H_2O)]^+ \rightleftharpoons \{[(en)Pt(UH\text{-}N^1,N^3)]_4\}^{4+} + 4\,H_2O$$

As evident from $^1H$ NMR solution studies, there are two conformers in equilibrium, which are analogues of cone and 1,3-alternate forms of calix-[4]-arenes (Fig.3).

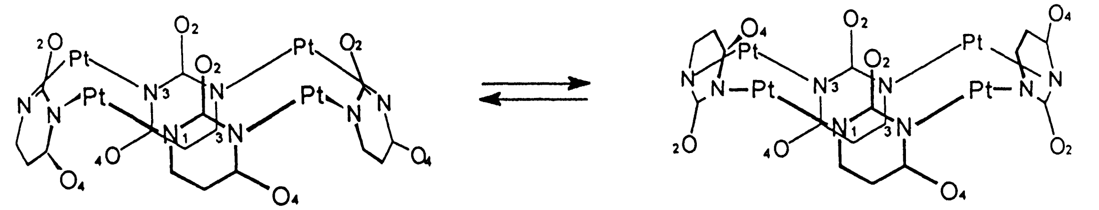

*Fig.3: Equilibrium between 1,3-alternate and cone conformer of $\{[(en)Pt(UH\text{-}N^1,N^3)]_4\}^{4+}$.*

The existence of both conformers was established by crystal structure analysis. The solid state structure of $\{[(en)Pt(UH\text{-}N^1,N^3)]_4\}^{4+}$ represents the 1, 3- alternate conformer. An additional example of the 1, 3-alternate form is realized in the heteronuclear $Pt_4$ / $Ni_4$-octamer, where Ni is bound through pairs of O(2) and O(4). The cone conformer, however, is realized in the form of a $Pt_4$ / $Ag_4$ octamer. Within this structure, all O(2)s are on the same side of the $Pt_4$-plane. Four Ag-atoms are coordinated to these oxygens, which simultaneously link two Ag centers each.

**Towards cyclic systems with trans-$a_2$Pt(II) and / or mixed geometries**

Another area of research in our group is the development of a new strategy for the synthesis of metallamacrocycles of nucleobases in a controlled fashion. Such a synthesis would permit the design of well defined molecular receptors for a variety of guests. Basic idea hereby is to use di- and trinuclear metal nucleobase compounds as precursors (Fig.4). These starting compounds can be connected by reaction with additional bidentate ligands and formation of cyclic units (Fig.5). The features of such receptors can be tuned by using ligands of suitable size and / or ligands with functional groups. Figure 4 shows the most simple example of such a starting compound. It is a $N^1,N^7$-diplatinated 9-methyladenine (9-MeA). Remarkable in this structure is the fact that the Pt-N vectors form an angle of about 90°. This fact suggests that it should be possible to arrange four purines in a coplanar fashion, linked by four metals with a linear coordination geometry. At the same time this starting complex is also a good precursor for the preparation of metal-modified nucleobase triples, which in turn are suitable intermediates on the way to larger cyclic systems (*vide infra*). Three feasible ways of synthesis are given in Fig.5. The ring size and ring shape is dependant on the ligands selected and the starting complex. Pure trans-compounds, either di-or trinuclear, with bridging purine entities are used as precursors in (A) and (B). Method (C) shows another possibility to control the ringshape. Here a trans-cis-trans complex is used as starting material.

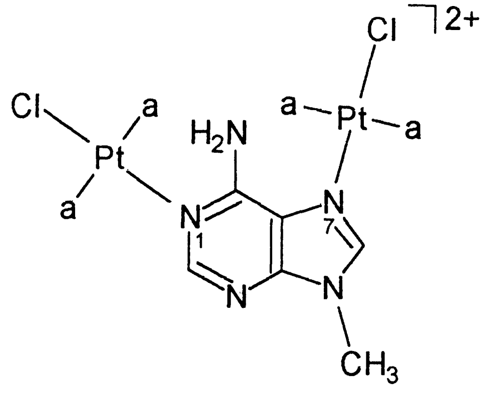

*Fig.4: trans-$[(Cla_2Pt)_2(9\text{-}MeA\text{-}N^1,N^7)]^{2+}$*

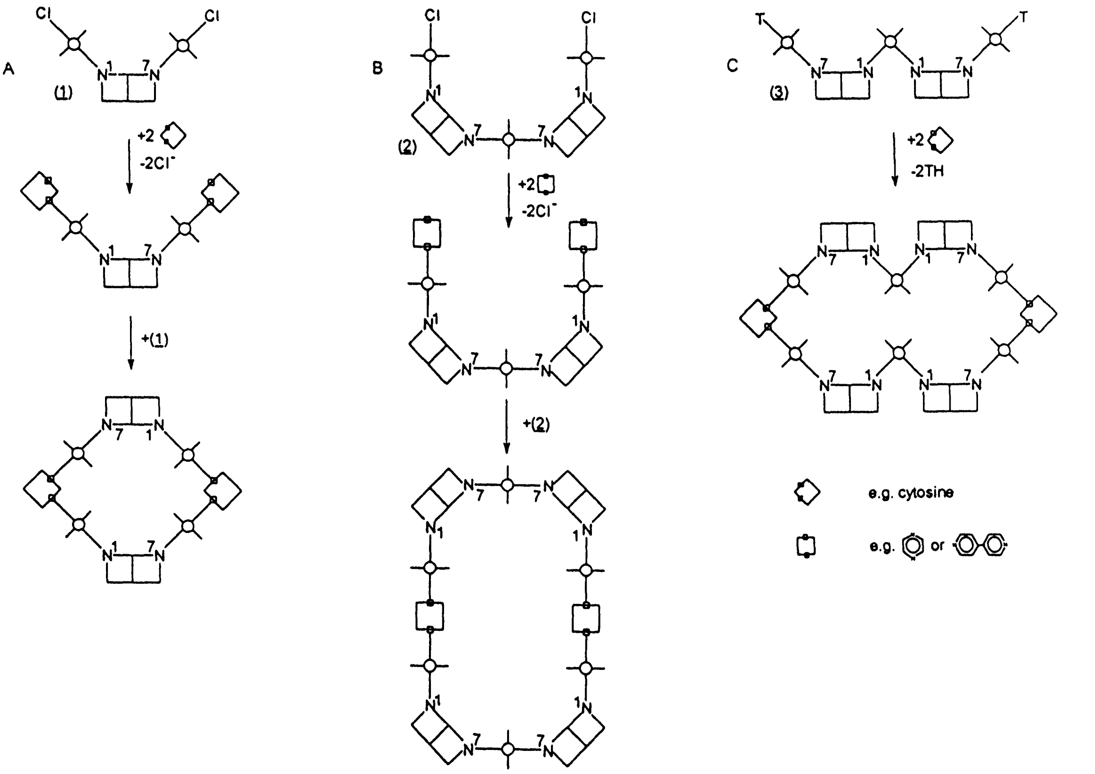

*Fig. 5: Three different possibilities for metalamacrocycle synthesis.*

The reaction pathways (A) and (B) are very similar. Both can proceed via triple, and quartet intermediates, respectively. On our way to cyclic compounds according to pathway (A), we have obtained a number of metal modified nucleobase triples. These complexes combine Watson-Crick and Hoogsteen base pairing schemes and are useful models of metalated forms of naturally occurring triple helices. Any combination of bases is possible, even formation of mispairs such as TAC or GAG. So far we have prepared two types of base triples of composition (py-pu-py) and (pu-pu-pu) ,respectively. In both cases the central purine is 9-MeA. Using unsubstituted pyrimidine ligands such as cytosine (Cyt), it is possible to prepare intermediates according to (A)1a in Fig.5. Here the formation of different products due to linkage isomerism (N(1),N(3) of Cyt) seems to be potentially complicating. However, applying $^{195}$Pt-editing spectral techniques, the two cytosines in our Cyt-(9-MeA)-Cyt compound can be shown to be bound exclusively through N(3).

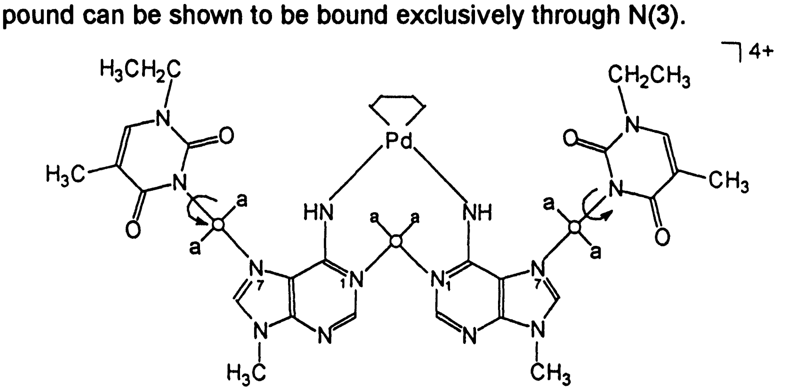

*Fig. 6: cis-{[trans-($(NH_3)_2$(1-EtT)Pt($N^7$-9-MeA-$N^1$)]$_2$Pt$(NH_3)_2$}$^{4+}$*

Following reaction pathway (C), we have been able to synthesize the intermediate cis-{[trans-$((NH_3)_2(1\text{-}EtT)Pt(N^7\text{-}9\text{-}MeA\text{-}N^1)]_2Pt(NH_3)_2\}(ClO_4)_4$ (with 1-EtT=1-ethylthymine anion). In this case, as well as in (A) and (B), the requirement for a ring closing reaction is a head / head arrangement of the bases. Therefore it is necessary to "freeze" rotation about the Pt-N bonds. In the case of the $N^1$-coordinated trans-cis-trans precursor, a simple (en)Pd(II) entity, which is bound through the exocyclic functional amino groups of 9-MeAs, keeps the whole structure rigid (Fig.6).Subsequent removal of the terminal $EtT^-$ ligands and simultaneous reaction with suitable ligands should lead to ring closure.

### Conclusions

Multinuclear metal nucleobase complexes are of considerable interest with regard to the following topics:

(i) They nicely examplify metal binding patterns to the multifunctional nucleobases.

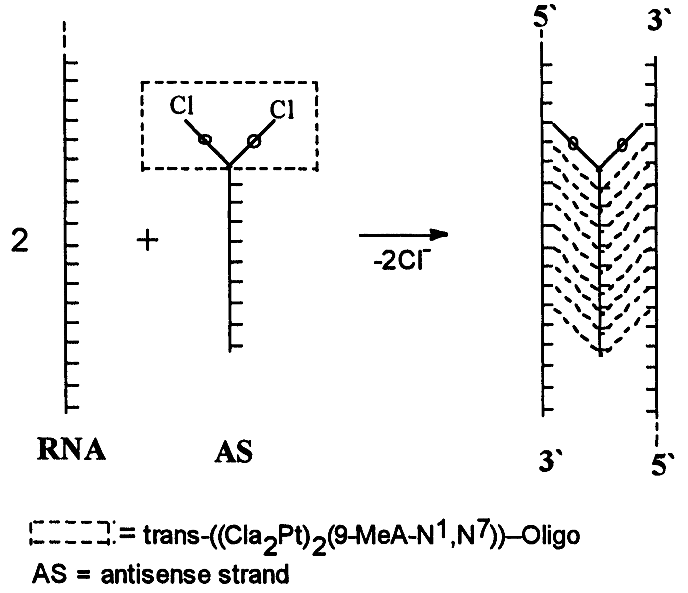

*Fig.8: Function of dimetalated purine entities in oligonucleotides as linking units.*

(ii) Cyclic nucleobase complexes represent novel examples potentially useful for host-guest studies.

(iii) Dimetalated purine entities in oligonucleotides might be useful reagents to accomplish triple strand formation with two target sequences (Fig.8). Possible applications in antisense and antigene strategies can be foreseen.

Work in our group is underway to explore these aspects in more detail.

### Acknowledgements

This work has been supported by the Deutsche Forschungsgemeinschaft (DFG) and the fonds der Chemischen Industrie (FCI).

### Literature

(1) See, e.g. (a) Lusty, J.R. (ed) *CRC Handbook of Nucleobase Complexes*, CRC Press: Boca Raton (1990), Vol.I; (b) Lusty, J.R.; Wearden, P.; Moreno, V. (ed), *CRC Handbook of Nucleobase Complexes*, CRC Press: Boca Raton (1992), Vol.II.

(2) See, e.g. (a) Hubert,J.; Beauchamp, A.L., *Can.J.Chem.*, **58**, 1439 (1980). (b) Charland, J.-P.; Simard, M.; Beauchamp, A.L., *Inorg.Chim.Acta*, **80**, L57 (1983).

(3) Lippert, B., Prog.*Inorg.Chem.*, **37**, 1 (1989).

(4) (a) Gagnon, C.; Beauchamp, A.L., *Inorg.Chim.Acta*, **14**, L52 (1975); (b) Gagnon, C.; Beauchamp A.L., *Acta Cryst.*, **B33**, 1448 (1977).

(5) (a) Farrell, N.; Qu, Y., *J.Am.Chem.Soc.*,**113**, 4851 (1991); (b) Farrell, N.; de Almeida, S.G.; Skov, K.A., *J.Am.Chem.Soc.*, **110**, 5018 (1988);(c) Johnson et al., *Nuc.Acids Res.*, **20**, 1697 (1992).

(6) Guay, F.; Beauchamp, A.L., *J.Am.Chem.Soc.*, **101**, 6260 (1979).

(7) Aoki, K.; Saenger, W., *Acta Cryst.*, **C40**, 775 (1984).

(8) Dieter, I.; Lippert, B.; Schöllhorn, H.; Thewalt, U., *Z.Naturforsch.*,**45b**,, 731,(1978).

(9) (a) Authier-Martin, M.; Hubert, J.; Rivest, R.; Beauchamp, A.L., *Acta Cryst.*, **B34**, 273 (1978) (b) Menzer, S.; Hillgeris, E. C.; Lippert, B., *Inorg. Chim. Acta*, in press.

(10) Krumm, M.; Zangrando, E.; Randaccio, L.; Menzer, S.; Danzmann, A.; Holthenrich, D.; Lippert, B., *Inorg.Chem.*, **32**, 2183 (1993).

(11) Krumm, M.; Lippert, B.; Randaccio, L.; Zangrando, E., *J.Am.Chem.Soc.*, **113**, 5129 (1991).

(12) Krumm, M.; Zangrando, E.; Randaccio, L.; Menzer, S.; Lippert, B., *Inorg.Chem.*, **32**, 700 (1993).

(13) Rauter, H.; Hillgeris, E.C.; Lippert, B., *J.Chem.Soc., Chem.Commun.*, **19**, 1385 (1992).

(14) Rauter, H,; Hillgeris, E.C.; Erxleben, A.; Lippert, B., submitted to *J.Am.Chem.Soc.*

**Received: September 29, 1993**

